# Histopathological Tumor and Normal Tissue Responses after 3D-Planned Arc Radiotherapy in an Orthotopic Xenograft Mouse Model of Human Pancreatic Cancer

**DOI:** 10.3390/cancers13225656

**Published:** 2021-11-12

**Authors:** Sophie Dobiasch, Severin Kampfer, Katja Steiger, Daniela Schilling, Julius C. Fischer, Thomas E. Schmid, Wilko Weichert, Jan J. Wilkens, Stephanie E. Combs

**Affiliations:** 1 Department of Radiation Oncology, Technical University of Munich (TUM), Klinikum rechts der Isar, Ismaninger Straße 22, 81675 Munich, Germany; severin.kampfer@mri.tum.de (S.K.); daniela.schilling@tum.de (D.S.); julius.fischer@tum.de (J.C.F.); thomas.schmid@helmholtz-muenchen.de (T.E.S.); wilkens@tum.de (J.J.W.); stephanie.combs@tum.de (S.E.C.); 2 Institute of Radiation Medicine (IRM), Department of Radiation Sciences (DRS), Helmholtz Zentrum München, 85764 Neuherberg, Germany; 3 Deutsches Konsortium für Translationale Krebsforschung (DKTK), Partner Site Munich, 80336 Munich, Germany; wilko.weichert@tum.de; 4 Physics Department, Technical University of Munich (TUM), James-Franck-Str. 1, 85748 Garching, Germany; 5 Institute of Pathology, Technical University of Munich (TUM), Trogerstr. 18, 81675 Munich, Germany; katja.steiger@tum.de; 6 Comparative Experimental Pathology, Technical University of Munich (TUM), Trogerstr. 18, 81675 Munich, Germany

**Keywords:** image-guided high-precision radiation, stereotactic irradiation, histopathology, pancreatic cancer, preclinical tumor mouse model, small-animal radiation research platform (SARRP), translational research, survival after high-precision irradiation

## Abstract

**Simple Summary:**

Despite intense research, the prognosis of patients with pancreatic cancer remains very poor without significant improvements over the past years. Novel therapeutic approaches may contribute to overcome therapeutic resistance and improve clinical outcome. Recently, small-animal imaging and high-precision irradiation devices for preclinical tumor models have been developed. The present preclinical study aimed to assess the accuracy of small-animal imaging and high-precision radiotherapy (RT) in an orthotopic xenograft pancreatic tumor mouse model by histopathological examination of the DNA damage marker γH2AX. Immunohistochemical staining of the pancreatic tumor and abdominal organs confirmed the accuracy of outlining based on CBCT imaging as well as the subsequent stereotactic RT. A clinical example from a patient with pancreatic cancer demonstrates the translational nature of this study. Longitudinal follow-up after high-precision RT showed a significant survival benefit in comparison to untreated tumor-bearing control mice. This preclinical RT platform provides the ability to mimic contemporary human RT closely and to evaluate novel RT concepts for pancreas cancer treatment.

**Abstract:**

Pancreatic ductal adenocarcinoma (PDAC) is one of the most lethal human cancers. Innovative treatment concepts may enhance oncological outcome. Clinically relevant tumor models are essential in developing new therapeutic strategies. In the present study, we used two human PDAC cell lines for an orthotopic xenograft mouse model and compared treatment characteristics between this in vivo tumor model and PDAC patients. Tumor-bearing mice received stereotactic high-precision irradiation using arc technique after 3D-treatment planning. Induction of DNA damage in tumors and organs at risk (OARs) was histopathologically analyzed by the DNA damage marker γH2AX and compared with results after unprecise whole-abdomen irradiation. Our mouse model and preclinical setup reflect the characteristics of PDAC patients and clinical RT. It was feasible to perform stereotactic high-precision RT after defining tumor and OARs by CT imaging. After stereotactic RT, a high rate of DNA damage was mainly observed in the tumor but not in OARs. The calculated dose distributions and the extent of the irradiation field correlate with histopathological staining and the clinical example. We established and validated 3D-planned stereotactic RT in an orthotopic PDAC mouse model, which reflects the human RT. The efficacy of the whole workflow of imaging, treatment planning, and high-precision RT was proven by longitudinal analysis showing a significant improved survival. Importantly, this model can be used to analyze tumor regression and therapy-related toxicity in one model and will allow drawing clinically relevant conclusions.

## 1. Introduction

Pancreatic ductal adenocarcinoma (PDAC) is one of the most lethal human cancers and is expected to be the second-leading cause of cancer-related deaths in the United States by 2030 [1,2]. The overall 5-year survival rate among patients with PDAC is less than 10% and only modestly improved in recent years [3,4]. The poor survival can be attributed to aggressive tumor growth and high therapeutic resistance [5,6,7]. The treatment of pancreatic cancer is still an unsolved health problem in industrialized countries and should be evaluated by multidisciplinary consensus at tumor boards to guarantee a therapy at the highest scientific standards [8,9]. Complete surgical resection remains the only curative treatment option but is initially only available for less than 20% of the patients [10]. Previous clinical trials show that at least one-third of patients, predominantly with locally advanced but irresectable lesions, benefit from local radiotherapy (RT) [11]. Especially, the novel technique stereotactic body radiotherapy (SBRT) with high single doses achieves an excellent local control and a moderate toxicity rate [12,13]. Due to the clear dose–toxicity relationship, further preclinical investigations, as well as future prospective trials, are essential to introduce innovative therapeutic approaches for patients with pancreatic cancer [14,15].

Translational research is currently undergoing a revolution process due to two key developments: the availability of advanced tumor models with a more clinically relevant tumor environment and the availability of small-animal imaging and RT devices [16,17]. These technologies allow precise radiation targeting using onboard integrated image guidance, reflecting clinically advanced RT treatments in an experimental setting [18,19]. However, to date, only a few preclinical data provide the precision of imaging, positioning, and radiation treatment in mouse models comparable to patient treatments [20].

The present study aimed to establish and validate a PDAC model of high-precision RT, which directly reflects important aspects of clinical RT. The accuracy of small-animal imaging and high-precision RT was successfully evaluated by histopathological examination of the tumor tissue and organs at risk (OARs) and compared to a clinical example. Furthermore, a longitudinal follow-up validated the efficacy of high-precision RT by the determination of survival.

## 2. Materials and Methods

### 2.1. Orthotopic Tumor Model

All animal procedures were carried out in strict accordance with the recommendations in the Guide for the Care and Use of Laboratory Animals of the National Institutes of Health. The protocol was officially approved by German law for the Care and Use of Laboratory Animals and authorized by the regional government of Upper Bavaria, Germany (reference 55.2-1-54-2532-217-2015 from 13 April 2016). All surgical procedures were performed under anesthesia, and all efforts were made to minimize suffering.

As previously described, an orthotopic xenograft pancreatic tumor mouse model in 6-week-old immunosuppressed CD-1^®^ nude mice (Crl: CD1- Foxn1nu, Charles River Laboratories, Sulzfeld, Germany) was established [21,22]. The two different established human pancreatic carcinoma cell lines, Panc-1 (CRL-1469) and MiaPaCa-2 (CRL-1420), were obtained from American Type Culture Collection (ATCC, Manassas, VA, USA). 2.0 × 10^6^ cells of the Panc-1 or 1.5 × 10^6^ cells of the MiaPaCa-2 cell line were injected into the parenchyma of the pancreas. When tumors reached a median tumor volume of 90 mm^3^ after a tumor growth of three (MiaPaCa-2) to eight (Panc-1) weeks, treatment planning and irradiation were performed.

### 2.2. Anesthesia

Mice were immobilized with inhaled isoflurane anesthesia at a concentration of 1.5% with a 6% volume of oxygen as a carrier gas during the whole procedure of imaging, treatment planning, and irradiation. The flow was adjusted to the individual need of mice.

### 2.3. Imaging

Cone-beam computed tomography (CBCT) incorporated in the SARRP device was used for imaging, treatment planning, and image-guided RT. The X-ray source of the SARRP was operated at a voltage of 60 kV and a current of 0.8 mA. We injected 5 mL per 1 kg mice bodyweight of the iodine-containing contrast agent Imeron (Bracco Imaging GmbH, Constance, Germany) intravenously (iv) into one of the lateral tail vessels to improve the soft tissue contrast and optimize the definition of the tumor volume and OARs for treatment planning. CBCT imaging was performed immediately after iv injection of Imeron using 1440 projections and a voxel size of 0.115 × 0.115 × 0.115 mm^3^.

### 2.4. Treatment Planning and Irradiation

The preclinical treatment planning software “MuriPlan” (Xstrahl Ltd., Camberley, UK) with its superposition-convolution algorithm for dose calculation was used for treatment planning. Gross tumor volume (GTV) and six OARs (both kidneys, small bowel, stomach, spinal cord, and liver) were delineated slice by slice on the CBCT image [23]. The GTV was defined as the macroscopically visible tumor tissue. A safety margin of 2 mm surrounding the GTV was considered. The radiation was delivered at 220 kV and 13 mA with a dose rate of 2.6 Gy/min by the SARRP device.

In the following, the single-dose high-precision irradiation of the GTV technique will be denoted as SBRT. An image-guided single-dose SBRT of 25 Gy compound dose-to-water (to the isocenter, which was in the center of the GTV) was performed in a single-arc technique with a gantry rotation from 178° to −178° with a field size of 10 × 10 mm^2^ using a fixed collimator. In comparison to the high-precision RT, whole-abdomen irradiation with 25 Gy (normalized to the isocenter, which was in the center of the GTV) was delivered in AP/PA opposing field technique with a gantry angle of 0° and 180° using a variable collimator with a field size of 50 × 30 mm^2^.

### 2.5. Histopathological Procedure and Quantification

A total of 18 mice were included in the histopathological analysis: 6 non-irradiated, tumor-bearing control mice, a cohort of 6 mice, which were irradiated with single-dose high-precision irradiation of the GTV, mimicking the stereotactic body radiation therapy of humans, and a cohort of 6 mice, which received a whole-abdomen RT as described in 2.4. Mice were euthanized one hour after irradiation by cervical dislocation. The tumor tissue and all OARs were collected, fixed in 4% formalin for 48 h, and transferred to 70% ethanol followed by embedding in paraffin. Tissue blocks were sectioned in 2 μm slices and stained with eosin (eosin y-solution 0.5% aqueous) and hematoxylin (Mayer’s hematoxylin) according to standard protocols.

The primary monoclonal antibody Phospho-Histone H2A.X ((Ser139), (20E3) Rabbit #9718, Cell Signaling Technology, Danvers, Massachusetts, USA, 1:500 dilution) and the ready-to use BOND Polymer Refine Detection containing the anti-rabbit HRP labeled secondary antibody for visualization (DS9800, Leica Biosystems, Nussloch, Germany) were used for the immunohistochemistry (IHC). IHC was performed and standardized by the fully automated research staining machine BOND RX (Leica Biosystems, Nussloch, Germany) according to the manufacturer’s instruction.

All slides were scanned with a digital pathology slide scanner “Aperio AT2” and analyzed quantitatively with the software “ImageScope” (Leica Biosystems, Nussloch, Germany). Positive staining for γH2AX was defined for at least 50% γH2AX-positive cells. In addition, the bioimage analysis open-source software “QuPath” was used to validate and determine total cell numbers and γH2AX-positive cells [24].

### 2.6. Longitudinal Follow-Up and Survival after SBRT

To determine the effect of SBRT in comparison to the unirradiated, tumor-bearing control group, mice were monitored longitudinally by at least weekly follow-up using CBCT as described in 2.3. The survival of mice was analyzed using GraphPad Prism software (version 8.0.2, GraphPad software Inc., San Diego, CA, USA). The Kaplan–Meier method was used to determine time-to-event analysis. Evaluation of differences in survival curves between the control and irradiated groups was performed by log-rank (Mantel–Cox) test. A total of 52 mice were included in the survival analysis: 16 (MiaPaCa-2) and 9 (Panc-1) non-irradiated, tumor-bearing control mice and a cohort of 19 (MiaPaCa-2) and 8 (Panc-1) mice irradiated with 25 Gy SBRT.

### 2.7. Clinical Example

To demonstrate the reflection of the clinical situation, imaging, treatment planning, and dose distribution of 25 Gy single-dose SBRT of a patient with PDAC treated at our department for radiation oncology is shown.

## 3. Results

### 3.1. Optimization of CBCT Imaging for Treatment Planning

**Figure 1:**
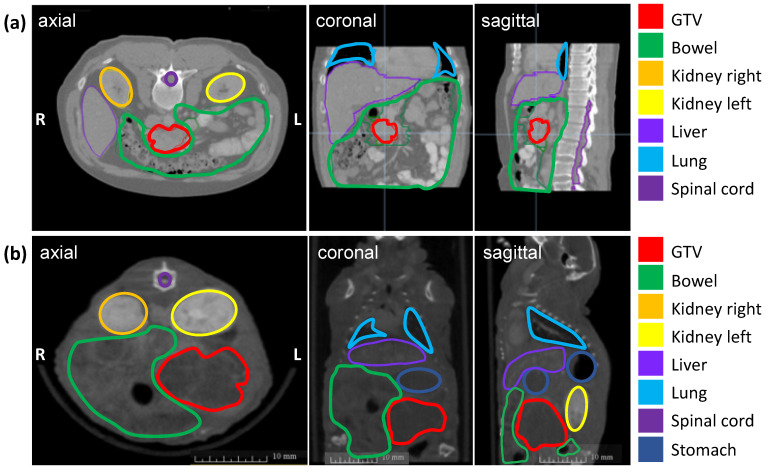
Planning imaging and contouring of the GTV (pancreatic tumor in red) and OARs (bowel in green, left kidney in orange, right kidney in yellow, the liver in lavender, lung in light blue, spinal cord in purple, and stomach in dark blue) in three different planes (axial, coronal, sagittal). (**a**) Planning CT images of a representative patient with PDAC; (**b**) Planning CBCT images of a representative mouse with an orthotopic pancreatic tumor.

The soft tissue contrast of all abdominal organs and the detection of the orthotopic tumor were optimized by iv injection of the iodinated contrast agent Imeron immediately before CBCT imaging and by an increase of the projections up to 1440. Figure 1 shows the definition of the GTV and OARs on an axial, sagittal, and coronal planning CT scan of a patient with PDAC (a) and a CBCT scan of a representative mouse with an orthotopic pancreatic tumor (b). The pancreatic tumor appears hypodense in CBCT imaging, the intestinal loops are partly air-filled, the kidneys in the retroperitoneum are contrasted strongly with Imeron, spine, and all other bony structures appear hyperdense. The iv contrast agent generally improves the soft-tissue contrast and enables the differentiation between all organs as well as the detection of the GTV due to uptake of Imeron by the kidneys, bowel, and all vessels.

### 3.2. Histopathological Analysis for Validating the Accuracy of High-Precision SBRT

The correct identification of the orthotopic tumor and the OARs by CBCT imaging and the accuracy of delivering high-precision RT to GTV were validated by γH2AX staining, the marker for DNA damage repair.

Histological slides of one representative mouse in each treatment group and the quantification of γH2AX-positive cells within a cohort are illustrated below.

#### 3.2.1. Proof of High-Precision Irradiation in an Orthotopic Pancreatic Tumor Model

**Figure 2:**
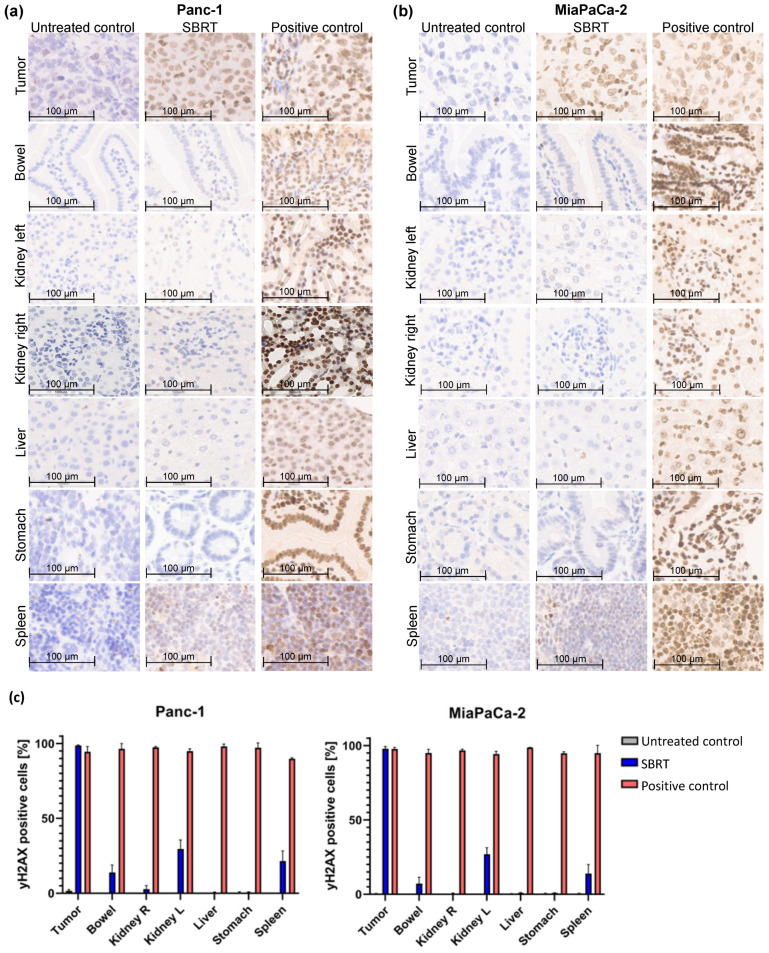
γH2AX staining by IHC and percentage of γH2AX-positive cells of different orthotopic pancreatic tumor tissues and OARs without RT (untreated control), one hour after SBRT, and whole-abdomen RT (positive control). (**a**) Representative orthotopic Panc-1 tumor tissue and OARs; (**b**) Representative orthotopic MiaPaCa-2 tumor tissue and OARs; (**c**) Quantification of the distribution of γH2AX-positive cells in the tumor tissue and OARs of all included mice.

To investigate the accuracy of high-precision SBRT in a clinically relevant orthotopic pancreatic tumor model, the γH2AX staining and percentage of γH2AX-positive cells were validated. Figure 2 shows the histological slides and quantification of the orthotopic tumor tissue, bowel, left kidney, right kidney, liver, stomach, and spleen after SBRT compared to the unirradiated tumor-bearing control group and those from mice after a whole-abdomen RT (positive control).

The group of the SBRT shows clearly that an intense γH2AX-positive staining (strong nuclear staining of γH2AX colored in brown) is observed only in tumor tissue, whereas the OARs show almost no staining (Figure 2a,b). In contrast, are the two control groups: as expected, no positive γH2AX staining is observed in the unirradiated group, and all tissues are stained γH2AX-positive in the positive group after whole-abdomen irradiation. The pancreatic tumor after SBRT reveals γH2AX staining comparable to the positive control. The distal OARs, including right kidney, stomach, and liver, show minimal to no γ-H2Ax staining after SBRT, similar to the untreated control group; instead, the cell nuclei are stained with the blue dye hematoxylin. Parts of the proximal OARs (bowel, left kidney and spleen) are located in the high-dose region and therefore show partly γH2AX-positive cells.

The γH2AX staining was validated by counting the total and γH2AX-positive cells of tumor tissues and OARs of all included mice. Almost 100% of γH2AX-positive cells are shown in pancreatic tumors after SBRT, comparable to the positive control. A mean value of 10%, 28%, and 16% γH2AX-positive cells was observed in the bowel, left kidney, and spleen after SBRT, respectively. The liver, stomach, and right kidney showed almost no γH2AX-positive cells after SBRT compared to the unirradiated control.

#### 3.2.2. Correlation of Treatment Planning with DNA Damage Activity

**Figure 3:**
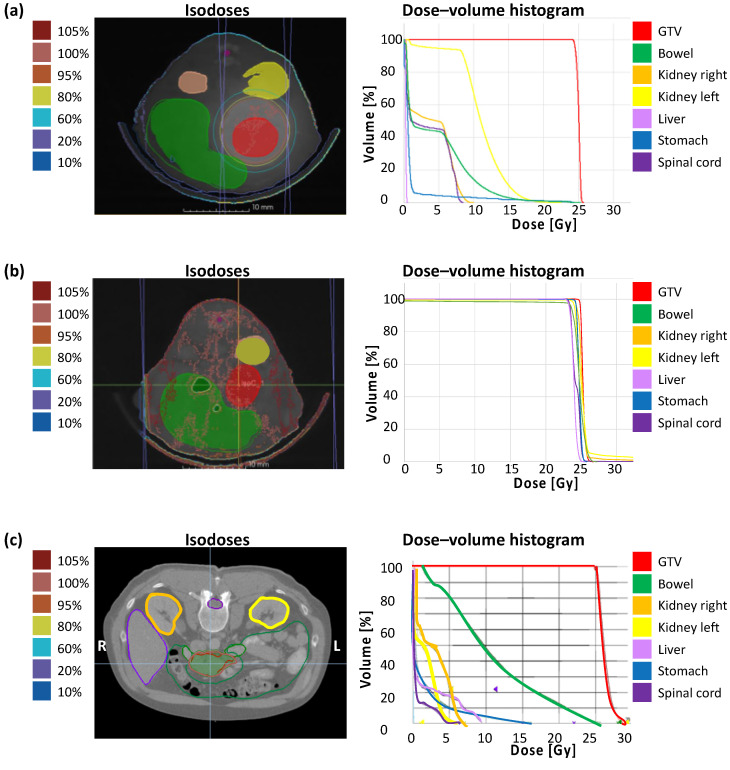
Treatment planning containing dose distribution with isodoses and dose–volume histogram. (**a**) Single-dose SBRT with 25 Gy in arc technique with a gantry rotation between 178° and −178° in vivo; (**b**) Wholeabdomen RT with 25 Gy in AP/PA technique in vivo; (**c**) Clinical example of a single-dose SBRT with 25 Gy in a PDAC patient.

The correlation of calculated dose distribution in GTV and OARs and the extent of the irradiation field by immunohistochemical staining of γH2AX in vivo are demonstrated in the following. To emphasize the translational significance, a clinical example with the planning parameters is included. Figure 3 shows the dose distribution with according isodoses and the dose–volume histogram (DVH) of a representative treatment plan of SBRT (a), unprecise whole-abdomen irradiation (b), and a clinical example (c). Performing whole-abdomen irradiation, almost 100% of the target volume and all OARs receive the prescribed dose of 25 Gy (Figure 3b). In contrast, the DVH of SBRT in vivo reveals optimal dose coverage of at least V95 > 95% in the GTV with simultaneous avoidance of OARs (Figure 3a), similar to the treatment plan of a patient with PDAC (Figure 3c).

In addition, dosimetric results of GTV and OARs including the mean dose D_mean_ (GTV, bowel, kidneys, liver, stomach), the minimum dose D_min_ (GTV), and the maximum dose D_max_ (GTV, bowel, kidneys, spinal cord) after treatment planning for SBRT confirm the irradiation of the entire tumor and general sparing of neighboring organs, and demonstrate the real translational approach (Table 1).

**Figure 4:**
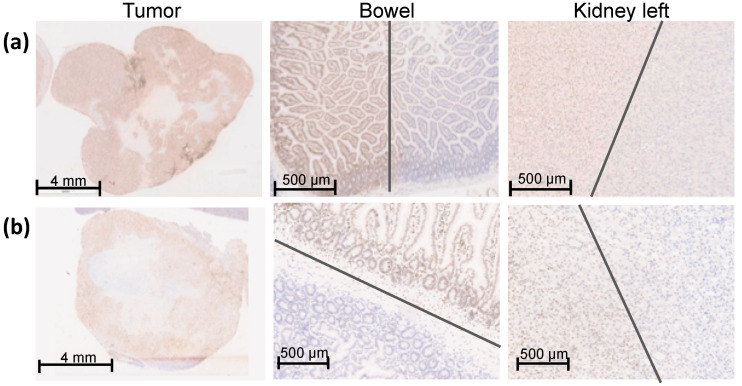
γH2AX staining of an overview of the entire orthotopic pancreatic tumor, bowel, and left kidney one hour after SBRT with 25 Gy. The entire pancreatic tumor tissue shows an intense γH2AX staining. The sharp gradient of the irradiation field is shown in both proximal OARS, the bowel, and the left caudal kidney, which were partly within the irradiation field. The grey lines mark the transition of the high-dose region to the low-dose region. (**a**) Representative Panc-1 tumor tissue and proximal OARs (bowel and left kidney); (**b**) Representative MiaPaCa-2 tumor tissue and proximal OARs (bowel and left kidney).

The dosimetric results from the planning analysis (Figure 3, Table 1) correlate with the DNA damage activity shown by γH2AX staining (Figure 4).

The entire tumor tissue was verified three-dimensionally by γH2AX staining and showed a homogeneous distribution of γH2AX staining in the whole tumor tissue. Thus, the complete dose coverage of the GTV by the used irradiation field was proved (Figure 4).

To determine the sharp gradient of the irradiation field, γH2AX staining in the proximate OARs is provided on a small scale. Figure 4 shows the bowel and the left kidney as proximate OARs at the edge of the high-dose region, which were partly within the high-dose irradiation field. Both organs received partially the maximal dose of 25 Gy comparable to the GTV, reflected by a positive γH2AX staining and no detectable biological damage outside of the high-dose region. The grey lines mark the transition of the high-dose region to the low-dose region.

A strong correlation between pre-calculated dose (Figure 3, Table 1) and DNA damage demonstrated by γH2AX staining (Figure 4) can be concluded.

### 3.3. Survival after SBRT

**Figure 5:**
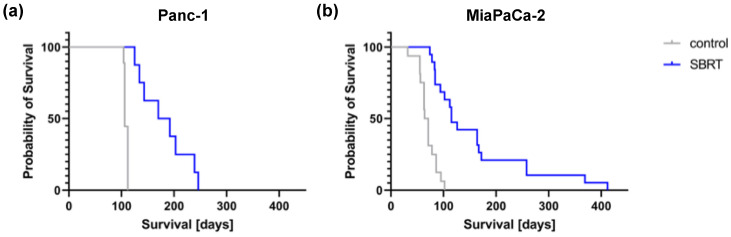
Kaplan–Meier survival curves after 25 Gy SBRT and unirradiated tumor-bearing control mice (*p* < 0.0001) after orthotopic injection of Panc-1 (**a**) and MiaPaCa-2 cells (**b**).

To validate the long-term effect of high-precision RT, mice were longitudinally monitored and the survival determined. Unirradiated Panc-1 and MiaPaCa-2 tumor-bearing mice showed a median survival of 106 and 67.5 days, respectively (Figure 5). A statistically significant improved median survival of 181 and 115 days was observed after SBRT with 25 Gy (*p* < 0.0001). The survival data confirm the histopathological findings and demonstrate the efficacy of 3D-planned high-precision irradiation in this clinically relevant mouse model.

## 4. Discussion

We developed an orthotopic mouse model for CBCT imaging and the establishment of high-precision RT concepts. The key findings of this paper are the proof of correct definition of the pancreatic tumor and the abdominal organs using CBCT imaging and the validation of the precision and accuracy of high-precision SBRT. For this goal, we included a total of 18 mice: 6 non-irradiated, tumor-bearing control mice, a cohort of 6 mice, which were irradiated with a single-dose high-precision SBRT, and a cohort of 6 mice, which received a whole-abdomen RT. Furthermore, the efficacy of high-precision RT was demonstrated by a significant increased survival in comparison to unirradiated control mice. To address the translational aspect of our preclinical model, a clinical example of a patient with PDAC was provided. Now, we can investigate the efficacy of different high-precision RT concepts in a mouse model and continue the research for personalized medicine in PDAC.

Xenograft tumor models in immunodeficient mice are the most commonly used animal models to assess novel therapeutic approaches. However, complex interactions including stromal cells, matrix proteins, endothelia, immune cells, and neighboring epithelial cells are interrupted [25]. The limitation of these immunodeficient models is an incomplete tumor microenvironment as immune infiltrates play an important role in shaping the tumor microenvironment and the disease [26]. Furthermore, the survival after irradiation might be affected, as SBRT contributes to an antitumor immune response by necrotic cell death and influences the immune profile [27].

CBCT imaging, treatment planning, and irradiation were performed by the SARRP system with its well-suited technology for small animal models equivalent to clinical high-precision RT [28,29]. We established a high-precision RT of an orthotopic pancreatic tumor mouse model with a good dose coverage of the GTV and sparing of the neighboring OARs using onboard CBCT imaging. Validation of the tumor and normal tissue with the DNA damage repair marker γH2AX one hour after irradiation confirms the exact delivery of the irradiation beam and the extension of the RT field.

Recently, Verhaegen et al. published an overview of technology for precision small animal RT research as well as its optimal use and challenges. Specific challenges include target motion, optimal irradiation margins, accuracy and precision of small field dosimetry, and methods to verify the dose distribution. In addition, “ESTRO’s Advisory Committee in Radiation Oncology Practice” recommends developing protocols and guidelines to use the novel preclinical radiation research platforms to maximize their impact on translating preclinical RT research into the clinic [18]. Our present study addresses a few mentioned challenges and shows an example for a smooth workflow of SBRT in an orthotopic pancreatic tumor mouse model. In addition, histopathological strategies to verify the accuracy of irradiation and dose distribution are provided, and a solid basis for subsequent translational research was developed.

First, important key experiments in small animal precision RT research were performed by Thorek et al. [30]. A radiopaque iodinated contrast agent was applied intraperitoneally to detect orthotopic pancreatic cancer models and abdominal organs by a separate X-ray CT. The precision of X-ray RT was confirmed by γH2AX staining. Their results allow the evaluation of tumor progression and therapeutic response in preclinical models.

The advantage and innovation of our present study is the use of CBCT, which is incorporated in the small animal irradiation device, for imaging and treatment planning. Although magnetic resonance imaging provides superior soft-tissue contrast, the main advantages of CBCT imaging are the short acquisition times and neither movement of the mouse nor positioning errors of the target [31]. While the anatomy of the head remains relatively immobile, significant motion is observed within the abdomen [18] and presents a substantial challenge in pancreatic tumor models. We improved the CBCT imaging by using an iv contrast agent as well as by increasing the numbers of projections. Thus, we are able to detect the tumor and all abdominal organs by CBCT. The treatment planning and SBRT are performed immediately after CBCT imaging. Uncertainties due to positioning changes between different imaging modalities are obsolete, resulting in increased precision of the RT and a reduction of appropriate safety margins around target volumes. In addition, due to the improved tumor localization, the irradiation field can be reduced; thus, neighboring organs are exposed to less radiation dose, and the potential toxicity to normal tissue is minimized.

Another preclinical study established a bioluminescence imaging-guided irradiation in pancreatic tumor models. Tuli et al. [32] combined molecular bioluminescence imaging (BLI) and anatomic CBCT imaging for treatment planning. Therefore, BLI and CBCT images were coregistered by manual fusion, and orthotopic pancreatic tumors were irradiated using the SARRP. Tuli et al. were not able to identify the orthotopic pancreatic tumor by CBCT image; thus, the definition of the target volume was based only on BLI. Limitations of the study included using 2D optical imaging and offline methods for fusion to CBCT where the animal is transported between imaging modalities resulting in non-negligible uncertainties. In addition, a single orthogonally directed beam as a simple model of irradiating the tumor target was performed, whereas we used advanced rotational irradiation in our study [32]. Innovative improvements of BLI were recently published demonstrating a 3D bioluminescence tomography to guide treatment planning and irradiation in different orthotopic mouse models [33,34].

Our results provide an essential basis for other subsequent translational studies since we proved the accuracy of high-precision RT by CBCT imaging and showed statistically improved survival after high-precision RT. Thus, it offers a highly relevant tool for all further preclinical RT studies of pancreatic cancer, including advanced tumor models like genetically engineered or patient-derived xenograft mouse models, for an improved individualization of cancer therapy.

## 5. Conclusions

Our present study demonstrates the establishment of a high-precision RT performed by the SARRP device in a clinically relevant orthotopic pancreatic tumor mouse model.

This preclinical RT platform provides the ability to mimic contemporary human RT closely and to evaluate potential novel RT concepts for pancreas cancer treatment. Importantly, this model can be used to analyze tumor regression and therapy-related toxicity in one model and will allow drawing clinically relevant conclusions.

## Figures and Tables

**Table 1:** Dosimetric analysis of 25 Gy single-dose SBRT in vivo, whole-abdomen irradiation with 25 Gy in vivo, and a clinical example of SBRT with 25 Gy of a PDAC patient including the mean dose (D_mean_), the minimum dose (D_min_), and the maximum dose (D_max_) of the GTV and OARs.

Contours	SBRT Dose [Gy]	Positive Control Dose [Gy]	Clinical Example Dose [Gy]
GTV D_mean_	25.0	25.3	26.3
GTV D_min_	23.9	23.9	24.2
GTV D_max_	25.7	26.8	29.4
Bowel D_mean_	4.5	24.4	10.8
Bowel D_max_	25.3	27.3	27.7
Kidney left D_mean_	11.1	25.2	1.7
Kidney left D_max_	22.5	40.2	5.8
Kidney right D_mean_	3.9	25.2	2.7
Kidney right D_max_	9.9	40.6	7.1
Liver D_mean_	0.3	24.0	0.6
Spinal cord D_max_	8.5	39.2	8.6
Stomach D_mean_	1.2	24.7	1.7

## Data Availability

Data are contained within the article.
